# Telemedicine-Based Risk Program to Prevent Falls Among Older Adults: Protocol for a Randomized Quality Improvement Trial

**DOI:** 10.2196/54395

**Published:** 2024-03-26

**Authors:** David B Rein, Madeleine E Hackney, Yara K Haddad, Farah A Sublett, Briana Moreland, Laurie Imhof, Cora Peterson, Jaswinder K Legha, Janice Mark, Camille P Vaughan, Theodore M Johnson II, Gwen Bergen, Emory STEADI Implementation team

**Affiliations:** 1 Department of Public Health NORC at the University of Chicago Atlanta, GA United States; 2 Department of Medicine, Division of Geriatrics and Gerontology Emory University School of Medicine Atlanta, GA United States; 3 Atlanta Veterans Affairs Center for Visual & Neurocognitive Rehabilitation Atlanta, GA United States; 4 Birmingham/Atlanta VA Geriatric Research Education Clinical Center Atlanta, GA United States; 5 Department of Rehabilitation Medicine Emory University School of Medicine Atlanta, GA United States; 6 National Center for Injury Prevention and Control Centers for Disease Control and Prevention Atlanta, GA United States; 7 Department of Health Sciences NORC at the University of Chicago Chicago, IL United States; 8 School of Nursing and Health Professions University of San Francisco San Francisco, CA United States; 9 Department of Family and Preventive Medicine Emory University School of Medicine Atlanta, GA United States; 10 Department of Medicine, Division of General Internal Medicine Emory University School of Medicine Atlanta, GA United States; 11 See Acknowledgments

**Keywords:** aging, cost-effectiveness, elderly, fall risk screening, fall risk, falls, medication management, older adults, physical therapy, prevention, public health, telemedicine

## Abstract

**Background:**

The Center for Disease Control and Prevention’s Stopping Elderly Accidents, Deaths, and Injuries (STEADI) initiative offers health care providers tools and resources to assist with fall risk screening and multifactorial fall risk assessment and interventions. Its effectiveness has never been evaluated in a randomized trial.

**Objective:**

This study aims to describe the protocol for the STEADI Options Randomized Quality Improvement Trial (RQIT), which was designed to evaluate the impact on falls and all-cause health expenditures of a telemedicine-based form of STEADI implemented among older adults aged 65 years and older, within a primary care setting.

**Methods:**

STEADI Options was a pragmatic RQIT implemented within a health system comparing a telemedicine version of the STEADI fall risk assessment to the standard of care (SOC). Before screening, we randomized all eligible patients in participating clinics into the STEADI arm or SOC arm based on their scheduled provider. All received the Stay Independent screener (SIS) to determine fall risk. Patients were considered at risk for falls if they scored 4 or more on the SIS or answered affirmatively to any 1 of the 3 key questions within the SIS. Patients screened at risk for falls and randomized to the STEADI arm were offered a registered nurse (RN)–led STEADI assessment through telemedicine; the RN provided assessment results and recommendations to the providers, who were advised to discuss fall-prevention strategies with their patients. Patients screened at risk for falls and randomized to the SOC arm were asked to participate in study data collection only. Data on recruitment, STEADI assessments, use of recommended prevention services, medications, and fall occurrences were collected using electronic health records and patient surveys. Using staff time diaries and administrative records, the study prospectively collected data on STEADI implementation costs and all-cause outpatient and inpatient charges incurred over the year following enrollment.

**Results:**

The study enrolled 720 patients (n=307, 42.6% STEADI arm; n=353, 49% SOC arm; and n=60, 8.3% discontinued arm) from September 2020 to December 2021. Follow-up data collection was completed in January 2023. As of February 2024, data analysis is complete, and results are expected to be published by the end of 2025.

**Conclusions:**

The STEADI RQIT evaluates the impact of a telemedicine-based, STEADI-based fall risk assessment on falls and all-cause health expenditures and can provide information on the intervention’s effectiveness and cost-effectiveness.

**Trial Registration:**

ClinicalTrials.gov NCT05390736, http://clinicaltrials.gov/ct2/show/NCT05390736

**International Registered Report Identifier (IRRID):**

RR1-10.2196/54395

## Introduction

Falls were the leading cause of nonfatal injuries and the leading cause of unintentional injury-related deaths among older adults (aged ≥65 years) in the United States in 2020, resulting in approximately 37,000 deaths, 3 million emergency department visits, and 1 million hospitalizations [1]. Falls among older adults are expensive, resulting in US medical costs of US $50 billion in 2015 [2]. With the aging population, the burden of falls on health care systems will continue to rise if fall prevention efforts are not expanded in clinical settings [3]. In a systematic review, Gillespie et al [4] found that multifactorial interventions significantly reduced the rate of falling among community-dwelling older adults. In more recent years, digital and telehealth programs for older adults have been implemented to evaluate their effectiveness in improving overall health and fall prevention [5-7].

The Stopping Elderly Accidents, Deaths, and Injuries (STEADI) initiative, developed by the US Centers for Disease Control and Prevention (CDC), offers health care providers tools and resources to assist with fall risk screening and multifactorial fall risk assessment and interventions [8]. The core components of STEADI are to screen, assess, and intervene: (1) screen older adults for fall risk using the 3 key questions (Have you fallen in the past year? Do you feel unsteady when walking or standing? and Do you have a fear of falling?) or the CDC’s Stay Independent screener (SIS) to identify patients at risk of falls [9]; (2) assess those who screened at risk for modifiable risk factors including gait and balance disturbances, medication risk, home hazards, orthostatic blood pressure, vision changes, concerns about feet and footwear, need for vitamin D supplementation, and comorbidities that increase fall risk [10]; and (3) intervene with evidence-based clinical and community strategies to reduce fall risk by addressing modifiable risk factors identified during the assessment [11,12]. STEADI has been implemented in full in primary care settings and in abbreviated versions in pharmacy and inpatient settings [9,13,14].

Evaluations of STEADI’s impact on outcomes has been limited. In a previous implementation of abbreviated STEADI in community pharmacies, Blalock et al [13] conducted a randomized controlled trial that compared the fall reduction impact of a pharmacist-led abbreviated medication management assessment implemented in 65 community pharmacies in North Carolina and usual care. The intervention successfully identified medication risks but found no statistically significant differences between the intervention and usual care groups in subsequent prescribing or fall outcomes during the observation period.

A recent multicenter clinical fall prevention study, the Strategies to Reduce Injuries and Develop Confidence in Elders (STRIDE), implemented a cluster-randomized trial of a nurse-administered multifactorial intervention in 86 primary care practices. The study compared the effects of a multifactorial intervention targeting modifiable risk factors to enhanced usual care [15]. They found that the intervention resulted in a significantly lower rate of a first self-reported fall injury compared to enhanced usual care, but no significant differences in rates of a first adjudicated serious fall injury, hospitalization, or death were found between the 2 groups [15]. Additional efforts are needed to evaluate the impact of clinical fall prevention on various health outcomes in clinical settings, including through telemedicine.

This paper describes the implementation protocol of the STEADI Options Randomized Quality Improvement Trial (RQIT) as implemented through telemedicine in primary care clinics to reduce falls among community-dwelling older adults. We designed the STEADI Options RQIT to estimate the effectiveness and cost-effectiveness of the STEADI assessment when pragmatically implemented through telemedicine.

## Methods

### Study Design

**Figure 1:**
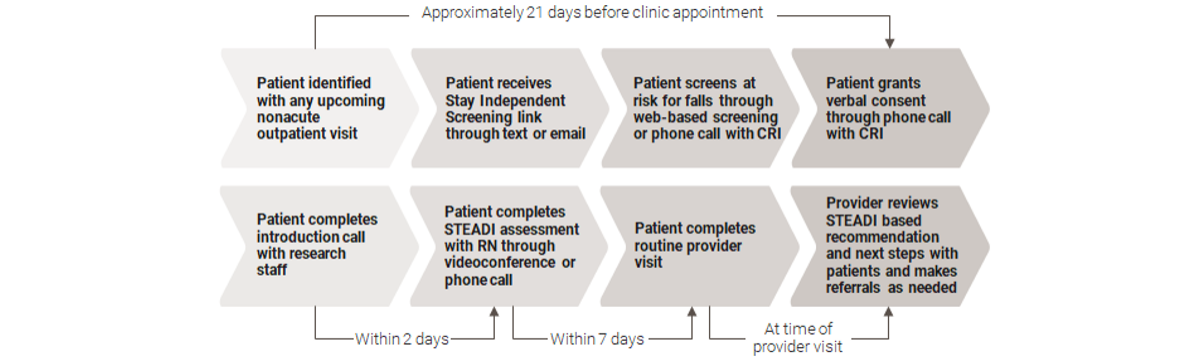
Clinical workflow of the Stopping Elderly Accidents, Deaths, and Injuries (STEADI) Options Trial: recruitment, screening, STEADI assessment, and intervention recommendation (Emory Health Services, 2020-2021). Those who screened at risk for falls but were assigned to the standard of care arm did not complete steps 5, 6, and 8. CRI: clinical research interviewers; RN: research nurses.

The STEADI Options RQIT was implemented in 5 primary care clinics between September 1, 2020, and December 31, 2021. The RQIT used a randomized design to compare patients at risk for falls who were assigned to receive a one-time telemedicine fall prevention assessment to a control group of patients at risk for falls who received the standard of care (SOC) of general disease state management. Due to the COVID-19 pandemic, screening was conducted through SMS text messaging–initiated surveys and phone calls, and we adapted the STEADI fall assessment to be implemented through phone- or video-supported telemedicine encounters conducted by a designated RN. Primary care providers were sent assessment information that they could access during the patient’s scheduled visit. We collected patient-level data on recruitment, assessments, health service use, prescriptions, fall events, implementation costs, and all-cause outpatient and inpatient costs incurred over one year using administrative and electronic health records (EHRs) and patient surveys. The following sections describe the study’s implementation and procedures (Figure 1).

The STEADI Options RQIT was a quality improvement initiative supported by health system leadership. Team members obtained verbal consent from study participants to share contact information with an evaluation partner outside the institution for the purpose of surveying (National Opinion Research Center [NORC] at the University of Chicago). Willingness to participate in the evaluation phase did not impact whether the patient received the STEADI evaluation as part of the quality improvement program.

### Ethical Considerations

The study protocol was reviewed and approved by the US Office of Management and Budget to assure its compliance with the Paperwork Reduction Act (Office of Management and Budget control number, 0920-1281). Evaluation aspects of the RQIT deemed to be human subjects research (patient surveys) were reviewed by the Emory Institutional Review Board to assure the safety and ethical treatment of human subjects (00111996), and the RQIT was registered (ClinicalTrials.gov NCT05390736).

### Study Setting and Staffing

We selected 5 primary care clinics based on the willingness of clinic leadership to participate, clinic patient population diversity, and the number of older adults served. The research team included representatives from CDC’s Division of Injury Prevention, NORC at the University of Chicago, and the Emory School of Medicine and Emory Healthcare primary care clinicians and staff. The Division of Injury Prevention was responsible for project oversight and fall prevention expertise, NORC designed the study protocol and data collection instruments and managed the project, and Emory was responsible for clinical implementation. Emory hired clinical research interviewers (CRIs), a clinical research coordinator, and RNs to conduct recruitment, coordination, and assessments. CRIs were responsible for screening patients for fall risk, recruiting and enrolling those at risk, scheduling STEADI assessments, and conducting preassessment coordination calls. The clinical research coordinator managed the workloads of the CRIs. The RN was responsible for conducting assessments, providing patient education, creating recommendations based on assessment information, and disseminating those recommendations to the patient's provider. Providers were responsible for determining whether to act on recommendations through the creation of clinical referrals to services or by managing medications. Emory implementation staff participated in monthly calls with the research team to discuss recruitment and implementation. Emory additionally convened monthly meetings between Emory implementation staff and primary care clinic staff to communicate the study design, intent, and responsibilities.

### Information Technology Infrastructure

Before the implementation, Emory integrated the SIS into Tonic (Tonic Solutions), an electronic platform used to administer previsit screening. Through Tonic, patients were able to take the SIS on a web-based device before their appointment using a phone, tablet, or computer, with the results automatically populating the patients’ EHR. The research team used REDCap (Research Electronic Data Capture; Vanderbilt University), a secure, internally hosted web-based application, to capture STEADI assessment results. The RN sent conclusions and recommendations to the provider using the EHR messaging center.

### Study Eligibility

Patients aged 65 years or older with a nonacute outpatient visit scheduled at participating primary care clinics were eligible for screening. Patients completed the SIS, and those with an SIS score of 4 or higher or those who answered “Yes” to any of the statements used as proxy to the 3 key questions within the SIS were eligible for enrollment (Textbox 1) [9]. Patients needed to be cognitively able to participate in the assessment, be able to converse in English, and have access to an able-bodied person to help with or respond to an emergency during the STEADI gait and balance assessments conducted by telemedicine (Textbox 1). Patients without valid phone numbers and those who opted out of receiving health system text messaging were excluded.

**Stopping Elderly Accidents, Deaths, and Injuries (STEADI) Options trial patient eligibility criteria, Emory Health Services, 2020-2021.**

**Patients aged 65 years or older with a qualifying (ie, nonacute) visit scheduled at 1 of the 5 designated Emory clinic implementation sites and:**

- Identified as high risk for falls through the Stay Independent screener by scoring 4 or more or answering affirmatively to any of the following statements in the Stay Independent screener as proxy to the 3 key questions:
  Sometimes I feel unsteady when I am walking.
  I am worried about falling.
  I have fallen in the past year.
- Able to participate in the STEADI assessments:
  Cognitively able to answer screening questions and participate in assessment.
  English-speaking.
  Have a computer, tablet, phone with internet and a webcam, or telephone.
  Have an able-bodied helper who can be available to help or called for help during the STEADI gait and balance assessment (in case of unsteadiness or a fall).

### Randomization

Providers were randomized each week 1:1 to either the STEADI or SOC arms based on a dice roll, and their scheduled patients were assigned to study arms based on the provider’s assignment. Providers were rerandomized each week; however, patient assignment remained the same even if they rescheduled an appointment to another week. During 3 weeks in the summer of 2021 when the RN was unavailable, all providers and their patients were assigned to the SOC arm. Over the subsequent 6 weeks, providers and their patients were assigned to the STEADI arm on a 2:1 basis to restore assignment balance, after which the study returned to 1:1 assignment. A total of 2 additional study arms with modified STEADI assessments were discontinued early in the study due to low overall enrollment.

### Screening, Recruitment, and Enrollment

Before their scheduled primary care visit, all eligible patients received a web-based or text link to a web-based SIS to complete. CRIs attempted to contact patients who had not completed the SIS on the web up to 3 times and completed the screener with them by phone if they were reached. Patients who screened at risk through the web-based SIS or through CRI contact and were randomized to the STEADI arm depending on the provider they were seeing were asked if they had (1) a computer, tablet, or phone with a camera and internet—if not, if they could participate by phone; (2) a clear hallway and corner in their home with space to assess gait or balance; and (3) someone nearby they could call upon for help if needed during the assessment. Those patients who answered yes to all 3 questions were then asked to provide informed consent to participate in the study. Patients who consented were asked if they had a blood pressure cuff at home (if not, the CRI ordered a cuff to be sent to their address), scheduled for a preassessment technology and home set-up call, and scheduled for a STEADI assessment before the primary care visit. Patients received an email with study information and instructions outlining the assessment process. Patients who were reached but did not consent to study inclusion were offered and provided STEADI assessments, although their data were not transmitted to the analytic team and were not included in analyses. Patients who were not reached were not included in analyses.

### Preassessment Technology and Home Set-Up Call

A total of 2 days before the STEADI assessment, CRIs called each patient and provided setup instructions based on a script. CRIs confirmed the patient had a stable internet connection and was able to start the videoconferencing software (Zoom; Zoom Video Communications). They asked the patient to use tape to mark 5 feet of space from their video call device for the visual acuity test and to mark a 10-foot path for the Timed Up and Go (TUG) physical therapy assessment. CRIs also had patients choose a safe corner of their homes for the modified 4-Stage Balance physical therapy assessment and ensured that the patient had an appropriate chair and wall space to set the chair against for the 30-Second Chair Stand physical therapy assessment. CRIs presented demonstration videos of STEADI’s 3 physical therapy assessments and helped patients adjust camera angles. Those patients scheduled for phone assessments were reminded of the scheduled assessment visit and given information regarding what to expect.

### STEADI Assessments

#### Overview

During the subsequent assessment call, the RN conducted 6 standardized assessments to identify the patient’s risk factors for falls. The protocol prioritized video calls but allowed for phone assessments for those unable or unwilling to participate in video assessments. Summaries of each assessment component, possible component findings, intended recommendations and interventions, and adaptations for phone assessments are described below and in table form (Multimedia Appendix 1).

#### Comorbidity Review

Before the assessment, the RN reviewed the patient’s EHR problem list for diagnoses of 6 comorbidities (Multimedia Appendix 2) associated with fall risk: cognition problems, Parkinson disease, cardiac arrhythmia, depression, or urinary incontinence [16,17]. Comorbidities were shared with providers in the RN’s recommendation statement.

#### Medication Review

Before the assessment, the RN reviewed the patient’s EHR medication list for any prescriptions from 10 classes of medications known to increase fall risk based on the Beers criteria (Multimedia Appendix 3) [18]. The RN additionally checked for polypharmacy, most commonly defined as prescriptions for 5 or more medications [19]. During the assessment, the RN confirmed with the patient the accuracy of the prescriptions listed in the EHR. The RN reported medication-related fall risk in her or his report for the provider and provided medication management educational materials to the patient (Multimedia Appendix 4). The RN also shared a recommendation to assess and adjust medications considered to increase risk of falls with providers.

#### Falls History

The RN asked how many times the patient had fallen in the past 12 months. If the patient reported a fall within the past 12 months, the RN asked if the patient sought medical attention for any fall or experienced loss of consciousness or broken or fractured bone or bones resulting from a fall. Fall history information was added to the provider report to increase the salience of fall prevention information.

#### Assessment of Feet or Footwear and Diabetes Assessment

The RN observed (through video) or asked about the patient’s current footwear, asked about foot pain or loss of sensation, and when applicable noted a diabetes diagnosis. Patient reports of foot pain, loss of sensation, or diabetes resulted in a RN note to the provider to examine the patient’s feet and the potential need for a referral to podiatry. The RN also reviewed a safe footwear handout (Multimedia Appendix 4) with all patients and emailed it to patients following the call.

#### Assessment of Visual Acuity

The RN projected the Banner eye chart on Zoom for the patient to read with their contact lenses or eyeglasses from five feet away (the distance was marked during the technology set up call) for both eyes together and each eye individually [20]. Phone patients were asked to self-report any vision problems. If the video screening or self-report indicated vision problems, the RN noted the result and recommended an eye health referral.

#### Gait and Balance

For video patients, the RN conducted the 30-Second Chair Stand test, the TUG, and the first three stages of CDC’s 4-Stage Balance Test [21-23]. During the 30-Second Chair Stand test, the RN counted the number of times in 30 seconds the patient rose to a full standing position from sitting in a chair without using their hands. For the TUG test, the RN timed how long it took a patient to stand up from their chair, walk 10 feet, turn around, walk back to their chair, and sit down. During the balance test, the RN observed the patient in 3 progressively difficult standing positions: (1) feet side-by-side, (2) one foot touching the big toe of the other foot, and (3) one foot in front of the other, heel touching toe. Patients assessed by phone without video were asked 10 questions (Multimedia Appendix 1) that corresponded with the physical domains tested by the 30 Second Chair Test, TUG, and the first three stages of 4-Stage Balance Test.

If a patient failed any of the mobility tests, exhibited signs of unstable gait, or reported difficulties, the RN discontinued gait and balance assessments and recommended physical therapy referral through the provider messaging center. With the increased popularity of fitness programs for older adults [24], the RN recommended a web-based tai chi for arthritis program (Multimedia Appendix 4) for patients that passed all 3 tests, as all patients were still considered to be at risk for falls based on the SIS [25].

#### Orthostatic Hypotension

The RNs evaluated observed changes in systolic blood pressure by measuring sit-to-standing blood pressure. Sit-to-standing was chosen over a supine-to-standing test to increase feasibility for in-home assessment [26]. The patient measured their blood pressure using their own cuff or one provided by the study and sat for at least 2 minutes before standing. Patients experiencing a systolic blood pressure drop of more than 15 mm Hg when standing from their chair were given STEADI-based educational materials on managing orthostatic hypotension (Multimedia Appendix 4), and the RN reported the finding to the provider for management [27]. The RN also asked the patient about dizziness and relayed any reports of dizziness to the provider.

#### Home Safety Risks

The RN reviewed the CDC brochure *Check for Safety: A Home Fall Prevention Checklist for Older Adults* (Multimedia Appendix 4) with each patient [28]. *Check for Safety* asks 17 questions about the home’s floors, stairs and steps, kitchen, bathrooms, and bedrooms and suggests ideas for removing or reducing fall hazards. The RN communicated home safety risks and a recommendation for occupational therapy to the provider for indicated patients.

#### Vitamin D Deficiency

Vitamin D deficiency is common and often underdiagnosed among older adults, although recommendations on supplementation are mixed [29]. The RN asked the patient if they usually take a vitamin D supplement with their other medications. If the patient did not take a vitamin D supplement, the RN recommended the provider check vitamin D levels and consider supplementation if vitamin D levels were less than 20 ng/ml.

#### RN Recommendations and Provider Action

The RN reviewed health education materials with the patient and sent these materials in an email follow up. RNs compiled assessment results, the educational materials presented to the patient, and their recommendations for referrals and care management and entered them into the patient’s EHR and sent assessment results and recommendations to the provider using the EHR messaging center. Providers in each participating clinic were informed about the study protocol, communication methods, and actions they could take to act on information contained in the RN report. Provider actions included ordering patient referrals to physical therapy, optometry, podiatry, the neurovestibular clinic (ie, dizzy clinic), and occupational therapy; reviewing and changing patient medications when indicated; and testing patients for vitamin D levels. Providers acted on RN recommendations at their clinical discretion (Multimedia Appendix 5). For example, at the patient’s upcoming primary care visit, providers would evaluate whether the medications identified by the RN should be adjusted or if a referral to Emory’s Dizziness and Balance Center and providing the Epley maneuver home exercise handout was warranted [30].

### Data Collection

To evaluate the intervention, we collected screening and assessment records, self-reported patient survey data, EHR health records, administrative medical cost information, implementation cost information through cost diaries, and qualitative interview data. Screening and assessment information was collected using a REDCap template. This included tracking compliance for the STEADI arm by recording whether each patient’s planned STEADI assessment occurred. Self-reported surveys were administered in 4 waves through web and phone follow up. We administered a baseline survey within 1 month of enrollment, and 3 follow-up surveys were administered at 4, 8, and 12 months after enrollment. The baseline survey collected patient information on fall perceptions, fall history, health status, and self-reported service and prescription use. Follow-up surveys collected information on fall events and on patient adherence to recommended treatments and STEADI-related service use (eg, physical therapy and occupational therapy). Survey respondents initially received a token of appreciation of US $3 in US postage stamps after completing each wave of the survey. After 13 months of implementation, we added a US $2 cash pre-incentive and a study-branded glasses cleaning cloth to increase response rates.

We extracted EHR and cost information from Emory records for each enrolled patient for the period of the participant’s enrollment date through 365 days of follow up. We also collected retrospective EHR and cost data for the 365 days before enrollment. We used time diaries completed by the CRI and RN to estimate implementation costs. We collected qualitative data from 45-minute phone interviews with the project RN and CRI and a sample of providers drawn from the Emory clinics implementing the study. All interviews were recorded and transcribed before being uploaded and coded in NVivo (Lumivero). A written summary of findings to contextualize quantitative data was developed.

### Evaluation

We will analyze process outcomes, short- and long-term outcomes, costs, and cost-effectiveness of the implementation (Multimedia Appendix 6). Process analyses will evaluate the extent to which assessments were conducted through phone or Zoom calls, evaluated intended risk factors, and identified risks among participants who were assessed. Qualitative interviews with providers will be thematically analyzed to provide details on whether the intervention was implemented as intended and provide additional information on intervention feasibility, communication between implementation staff, perceived efficacy, and patient engagement.

Using an intent-to-treat approach, we will evaluate sample balance and assess short- and long-term outcomes comparing the STEADI and SOC arms. We will use survey and EHR data to evaluate differences between the arms in the short-term outcomes of (1) STEADI-indicated service use (eg, physical therapy and occupational therapy) and (2) prescriptions for medications that increase fall risks. For the long-term outcomes of (1) medically treated and self-reported falls and (2) all-cause health care costs from the Emory Healthcare system, we will use EHR data and administrative cost data. We will use the *International Classification Definition, 10th Revision, Clinical Modification* (ICD-10-CM) codes (Multimedia Appendix 2) and chart text notes to identify medically treated falls at Emory. Preliminary analyses indicate that ICD-10-CM codes without text note review are insufficient to differentiate individual fall episodes or determine fall severity. Therefore, we will also use self-report of falling with treatment at Emory to guide additional text note reviews for fall events that may not have been coded in the EHR. Falls will be coded as EHR-confirmed, self-reported medically treated falls outside the Emory system (EHR not confirmed), and self-reported and not medically treated to support analyses. We will additionally evaluate the impact of STEADI using a per protocol approach using a 2-stage residual inclusion approach.

For cost and cost-effectiveness, using time diaries and programmatic records, we will estimate the total implementation cost of the intervention per person. Cost will be assessed both based on an intent-to-treat perspective per person and based on the per-protocol cost of implementation per person associated with full compliance with the study protocol. Using estimates of the implementation costs per person assessed and the intervention effect (if any) on falls and all-cause health expenditures, we will estimate the incremental cost-effectiveness of STEADI from the health care perspective. This will be compared to the SOC over one year using an incremental net benefit framework anchored to the willingness to pay to prevent 1 fall.

### Sample Size Estimation

We estimated a sample size based on the sample size required to detect a difference in the relative risk (RR) of falling at least once during the year between the STEADI arm and SOC arm. We assumed an absolute risk of any fall in the control group of 0.5 and a design effect of 1.05. We assumed a 50% risk of falling based on the expected performance of a screening tool to detect fall risk [31] and assumed a relatively small design effect under the assumption that individuals assessed were largely independent of each other in behavior given the telemedicine implementation. The results indicated that a sample of 500 in each arm would have 80% power of detecting an RR of 0.82, a sample of 350 would have 80% power of detecting an RR of 0.78, and a sample of 250 would have 80% power of detecting an RR of 0.75. Based on this, we sought to recruit at least 250 people in each arm with a recruitment goal of 500 per arm.

### Statistical Procedures

We will impute missing values in the survey data using multiple imputations by chained equations. Imputation will be conducted using logistic models for categorical variables and predictive mean matching for continuous and count variables. Analyses using imputed data will use methods that appropriately estimate standard errors for multiply imputed data. Quantitative analyses will include bivariate and multivariable logistic, Poisson, and linear regression models estimating the intent-to-treat effect of the STEADI initiative on fall outcomes. We will use prespecified multivariable statistical models to control for unbalanced covariates and reduce residual variance. Per-protocol estimates of the effect of STEADI on outcomes will be estimated using treatment assignment as an instrument for the STEADI risk screening and assessment. Finally, 2-part models [32] will be used to estimate health expenditures.

## Results

From September 1, 2020, to December 31, 2021, the study enrolled 720 patients, of whom 307 (42.6%) were assigned to the STEADI arm, 353 (49%) were assigned to the SOC arm, and 60 (8.3%) were assigned to arms that were discontinued. Process and implementation cost data were collected. Follow-up data collection was completed in January 2023. As of February 2024, data analysis is complete, and results are expected to be published in the end of 2025.

## Discussion

### Overview

The primary purpose of the CDC’s STEADI Options RQIT was to evaluate the impact of a telemedicine-based form of the STEADI fall risk screening and assessment on falls and all-cause health expenditures among older adults when implemented within a primary care setting. We hypothesize that intervention patients will experience fewer fall-related outcomes compared to the control group; however, this relationship may be challenging to detect. For example, the STRIDE study found that a multifactorial intervention was associated with an 8% reduction in the rate of a first adjudicated serious fall compared to an enhanced usual care, but this result was not statistically significant [33]. The STRIDE study considered fall injuries but not falls without injuries in their outcomes and required one source of independent adjudication to determine the primary outcome.

This study includes patient self-reports of falls that did not require medical attention and falls that resulted in medical attention obtained outside the Emory health care system. Sustaining a serious injury from a fall requiring medical care or serious injury requiring hospitalization or prolonged care is likely dependent on individual health and functional characteristics. Therefore, detecting differences in all falls, including those that did not require medical intervention, is important in understanding the potential impact of a fall prevention intervention. Further, our survey captures falls that required medical attention but were treated outside the Emory Healthcare System because patients may turn to alternative settings such as urgent care centers to treat injuries.

This study collects detailed data on the STEADI risk screening and assessment process, allowing us to measure implementation fidelity. We are also collecting data on short-term outcomes of service use and prescriptions for medications that increase fall risk. Having this level of detail will allow us to evaluate whether the intervention translated into patient use of preventive health services with the potential to prevent falls, even if no difference in falls is detected. Additionally, we collected time diary data on STEADI implementation to estimate the costs of replicating the intervention and the cost-effectiveness of the intervention.

### Limitations

Several factors limit this study. First, the COVID-19 pandemic required us to adapt STEADI from primary care, in-person risk screening, and assessment to telemedicine. As initially conceptualized, it was intended that all participants assigned to the intervention group would receive in-home video assessments through the Zoom platform. However, not all patients were able or willing to conduct assessments over video, so we adjusted the protocol to allow for some participants to be assessed by phone. We hypothesize that phone evaluations are less effective than video assessments because they replaced visual assessments of gait and balance, footwear, and vision problems with patient self-reported values likely underestimating risk. Ideally, the study design would have assigned patients to phone or Zoom assessments to compare outcomes by assessment type. However, the randomization of participants to phone or video calls was infeasible.

Second, conditions of the COVID-19 pandemic may have impacted the intervention’s effectiveness if patients avoided encounters with the health care system for social distancing reasons. Avoiding health care encounters would reduce use of fall preventive services and might result in a lower number of medically treated falls if patients deferred medical care for falls they would have sought care for in the absence of COVID-19. This limitation is likely to bias our results toward the null hypothesis of no intervention effect.

Third, the instance of the Emory EHR deployed at the time of the study did not track referrals. Therefore, if patients did not access services indicated by the assessment, we are unable to determine if this was due to a lack of provider referrals or low patient adherence. Further, a change in Emory’s EHR vendor resulted in a loss of approximately 6 weeks of follow-up data from 80 enrolled patients, a limitation we are adjusting for in statistical models. Fourth, not all patients completed each wave of the patient survey due to survey nonresponse. We will attempt to mitigate this limitation by using poststratification weights or imputation methods. Fifth, preliminary analyses have indicated that fall-related diagnosis codes in the EHR by themselves are limited in their ability to detect medically treated falls and differentiate unique fall events. We will attempt to mitigate this limitation using chart reviews but will likely miss some medically treated falls, biasing results toward the null. Sixth, because providers were randomized into STEADI and SOC arms by week, possible contamination in favor of fall-related interventions may have been introduced during the weeks providers were assigned to SOC, a limitation which would also bias results toward the null.

### Conclusion

Many older adult falls are preventable. The CDC’s STEADI initiative [10] offers tools and resources for health care providers to conduct clinical fall prevention efforts with their older patients. We designed this study to capture detailed information on the implementation, short-term outcomes, and long-term outcomes of STEADI fall risk assessment and screening when implemented through telemedicine through a pragmatic design in collaboration with a primary care setting. The main advantage of this study is the collection of self-reported fall outcomes and health service use information from the entire sample of participants. This study can provide evidence to support future implementations and adaptations of multifactorial fall prevention interventions such as STEADI.

## Appendix

Multimedia Appendix 1 Protocol for assessment of Stopping Elderly Accidents, Deaths, and Injuries (STEADI) Options Trial, Emory Health Services, 2020-2021.

Multimedia Appendix 2 International Classification of Diseases, Tenth Revision, Clinical Modification (ICD-10-CM) diagnosis codes indicating potential fall occurrences and comorbidities.

Multimedia Appendix 3 Stopping Elderly Accidents, Deaths, and Injuries (STEADI) Options Trial electronic record medication flag list for medication management assessment component.

Multimedia Appendix 4 Stopping Elderly Accidents, Deaths, and Injuries (STEADI) Options Trial educational materials distributed and reviewed to patients during assessment.

Multimedia Appendix 5 Collaboration between clinical research nurse (RN) and provider in the Stopping Elderly Accidents, Deaths, and Injuries (STEADI) Options Trial, Emory Health Services, 2020-2021.

Multimedia Appendix 6 Stopping Elderly Accidents, Deaths, and Injuries (STEADI) Options Trial evaluation design, Emory Health Services, 2020-2021.

## Abbreviations

CDC: Centers for Disease Control and Prevention
CRI: clinical research interviewer
EHR: electronic health record
ICD-10-CM: International Classification Definition, 10th Revision, Clinical Modification
NORC: National Opinion Research Center
REDCap: Research Electronic Data Capture
RN: registered nurse
RQIT: Randomized Quality Improvement Trial
RR: relative risk
SIS: Stay Independent screener
SOC: standard of care
STEADI: Stopping Elderly Accidents, Deaths, and Injuries
STRIDE: Strategies to Reduce Injuries and Develop Confidence in Elders
TUG: Timed Up and Go
