# Supplementary material for: Telemedicine-Based Risk Program to Prevent Falls Among Older Adults: Protocol for a Randomized Quality Improvement Trial
Source: JMIR Res Protoc. 2024 Mar 26;13:e54395. doi: 10.2196/54395 (PMC11005432; doi:10.2196/54395)
Supplement: Multimedia Appendix 1 [file resprot_v13i1e54395_app1.docx]

## Multimedia Appendix 1. Protocol for assessment of Stopping Elderly Accidents, Deaths, and Injuries (STEADI) Options Trial, Emory Health Services, 2020-2021.

| **STEADI-recommended Component** | **Assessment Used** | **Potential Findings** | **Recommended Intervention** | **Voice Call Adaptation** |
| --- | --- | --- | --- | --- |
| Falls history | | | |  |
| RN asked three questions about the patient’s fall history in the past year. | How many times have you fallen in the last year? | [1] Once  [2] Twice  [3] Three or more. | If patient has fallen more than once in the last year, the patient is at a serious risk for falls. RN will note increased provider urgency to mitigate risks. | No adaptation needed. |
|  | Did you seek medical attention as a result of any of these falls? | [1] No  [2] Talked to my doctor about it  [3] Went to the ER or was admitted to hospital. | If patient went to emergency room or was hospitalized, then RN will note increased provider urgency to mitigate risks. |  |
|  | Did you experience any of the following? | Check all that apply:  [1] Blackout/lost consciousness after the fall  [2] Break or fracture a bone as a result of falling  [3] None of the above. | If patient has experienced a blackout, lost consciousness, or broke/fractured a bone from a fall, then provider may consider further diagnostics to assess and address fall risk. |  |
| Assessment of feet/footwear and diabetes status | | | |  |
| RN asked the patient questions about feet and footwear and displayed the footwear handout via Zoom for review with the patient. After the assessment, RN emailed the handout. | Ask patient about what type of footwear they currently wear | [1] Risky (e.g., high feels, flip flops)  [2] Non-risky (e.g., low heel, firm sole, slip resistant). | Regardless of footwear, nurse reviews footwear handout with patient. | No adaptation needed for questions or chart review. RN will review feet and footwear handout verbally on voice call. |
|  | Ask patient about existing foot pain and loss of sensation. | Patient reports:  [1] Foot pain  [2] Loss of sensation  [3] Both  [4] Neither. | If patient has foot pain or loss of sensation and diabetes, provider examines feet and considers referral to podiatry. |  |
|  | Check chart/ask patient about diabetes status. | [1] Patient has diabetes  [2] Patient does not have diabetes. |  |  |
| Assessment of visual acuity | | | |  |
| RN asked patient whether they wear contact lenses or eyeglasses and if so, are they bifocals or progressives.  During the pre-assessment set-up call, CRIs assisted the patient with measuring a five-foot distance away from their computers. During the assessment, RN projected the Banner eye chart over Zoom and the patient stood five feet away for the test. | Record whether the patient wears contacts or glasses and if they are bifocal or progressive lenses. | [1] Glasses  [2] Contacts  [3] Neither.  [1] Bifocals  [2] Progressives  [3] Neither. | If patient has visual impairment (20/40 or worse), then ophthalmology or optometry referral is recommended. | Voice call patients are asked to self-report any vision problems. No Banner eye chart test is performed. |
|  | Record patient’s visual acuity in both eyes, as well as each eye individually, using Banner eye chart. | Visual acuity value. |  |  |
| Gait and balance | | | |  |
| The STEADI RN used falls history responses in combination with the Stay Independent Screener (SIS) to determine whether it was safe to proceed with the tests.  If RN deemed it safe, she conducted the chair stand test, followed by the Timed Up & Go (TUG) and then the 4-stage balance test. If a patient failed one test, RN may not have proceeded with subsequent tests. | Conduct 30-Second Chair Stand. | Number of times patient stands in 30 seconds.  If below any of the following cut-offs in the 30-second chair stand handout^1^ by age/sex then patient “failed” | If patient “fails” any of the Gait and Balance tests, physical therapy referral is recommended.  If the patient passes all three tests, patient is referred to online Tai Chi for Arthritis program.  If the patient exhibits signs of unstable gait, RN will note this in patient’s chart. | In lieu of the three tests, the following questions are asked:  1. Have you or anyone noticed that you’ve been walking at a slower pace?  [1] No  [2] Yes.  2. Have you or anyone else noticed any difficulties walking at your normal pace and/or your normal stride?  [1] No  [2] Yes.  3. Do you have to steady yourself on a wall while standing or while taking steps?  [1] No  [2] Yes.  4. Do you have difficulty turning?  [1] No  [2] Yes.  5. Do you need to push up from a chair to stand up?  [1] No  [2] Yes.  6. Would you be able to get up from the chair without using the arm rest or pushing off the chair in any way?  [1] No  [2] Yes.  7. Do you have any difficulties while standing?  [1] No  [2] Yes.  8. Can you stand with your feet side-by-side without any support?  [1] No  [2] Yes.  9. Can you stand with the instep of one foot touching the big toe of the other foot for 10 seconds?  [1] No  [2] Yes.  10. Could you stand with one foot directly in front of the other for about 10 seconds without any balance support or wobbling?  [1] No  [2] Yes. |
|  | Conduct Timed Up & Go (TUG) Test. | Did the patient exhibit signs of unstable gait, such as loss of balance, short strides, little or no arm swing, or en bloc turning during the assessment?  [1] No  [2] Yes |  |  |
|  |  | Number of seconds it takes for the patient to complete the test  If 12 or more, patient “failed” TUG. |  |  |
|  | Conduct Tandem Stance test. | Was the patient able to stand in tandem for 10 or more seconds?  [1] No- patient fails; number of seconds it takes for the patient to stand in tandem stance before losing balance  [2] Yes- patient passes. |  |  |
| Review of comorbidities | | | |  |
| RN reviewed the patient’s diagnoses and flagged whether they have specific selected conditions associated with fall risk. | Does the patient have one or more comorbidities associated with increased fall risk? | [1] No  [2] Yes  If yes, then select all that apply:  [1] Cognition problems (e.g., dementia)  [2] Parkinson’s  [3] Cardiac arrhythmia  [4] Depression  [5] Incontinence  [6] None. | If patient has a diagnosis flag or self-reports one of the five listed diagnoses, then patient has comorbidities associated with increased fall risk. Provider will use information to better manage medications. | No adaptation needed. |
| Medication review | | | |  |
| RN reviewed medications being taken by the patient using the SAFE Medication Review Framework^2^ and identified if patient is taking any fall risk-increasing medications. | RN reviews patient’s chart for current medications and asks patients what over-the-counter and prescription medications they are currently taking. | Select all medications that patient is taking:  [1] Tricyclic or other antidepressants  [2] Antihistamines/ allergy/cough and cold medicines  [3] Motion sickness/ dizziness/nausea medicines  [4] Anticholinergics  [5] Insomnia/sleep  [6] Gastrointestinal tract medications  [7] Anticholinergics or antipsychotics  [8] Skeletal muscle relaxants  [9] Benzodiazepines  [10] Z-drugs  [11] Opioids.  For those with orthostatic hypotension and/or dizziness, cardiac medications should also be reviewed | If any of the high-risk classes are checked, the provider will assess whether medications can be tapered or adjusted based on prescribing guidance.  RN provides patient-facing medication management materials based on which medications they are taking. | No adaptation needed. |
|  |  | Number of medications  If five or more, categorize as polypharmacy. | If polypharmacy indicated, provider will consider whether it is possible to de-prescribe. |  |
| Assessment of orthostatic hypotension and dizziness | | | | |
| During the pre-STEADI assessment calls, CRI ascertained whether patients have a blood pressure cuff. If they did not, then a blood pressure cuff was sent to the patient’s home. Patient recorded their own blood pressure during sit-to-stand test. | RN assesses orthostatic hypotension using the sit-to-stand test. | If change in systolic BP 15 or more then patient has orthostatic hypotension. | If patient has orthostatic hypotension, they will receive the [STEADI brochure on managing orthostatic hypotension](https://www.cdc.gov/steadi/pdf/STEADI-Brochure-Postural-Hypotension-508.pdf).  Provider considers how to adjust medications to mitigate orthostatic hypotension. | No adaptation needed. |
|  |  | [1] Patient reports dizziness during test  [2] Patient does not report dizziness during test. | If patient has dizziness, provider may consider conducting a medication review, referring patient to Emory’s dizzy clinic, and distributing a home exercise handout (Epley maneuver handout). |  |
| Home safety risks | | | |  |
| RN reviewed the CDC Check for Safety fall prevention checklist with patient. | Does the patient have home safety risks? | [1] No  [2] Yes | The STEADI nurse may recommend changes to reduce home hazards as well as a referral to occupational therapy based on checklist results. | No adaptation needed. |
| Vitamin D deficiency | | | |  |
| RN assessed potential vitamin D deficiency. | Do you take vitamin D normally with your other medications? | [1] No  [2] Yes. | For all patients, risk assessment summary will note vitamin D deficiency as a general fall risk factor. Provider may consider whether to supplement if the patient is deficient. | No adaptation needed. |

**Abbreviations:** STEADI, Stopping Elderly Accidents, Deaths, and Injuries; RN, clinical research nurse; CRI, clinical research interviewers
^1^30-Second Chair Stand assessment accessed via <https://www.cdc.gov/steadi/pdf/STEADI-Assessment-30Sec-508.pdf>

^2^SAFE Medication Review Framework accessed via <https://www.cdc.gov/steadi/pdf/steadi-factsheet-safemedreview-508.pdf>
