# Supplementary material for: Telemedicine-Based Risk Program to Prevent Falls Among Older Adults: Protocol for a Randomized Quality Improvement Trial
Source: JMIR Res Protoc. 2024 Mar 26;13:e54395. doi: 10.2196/54395 (PMC11005432; doi:10.2196/54395)
Supplement: Multimedia Appendix 2 [file resprot_v13i1e54395_app2.docx]

## Multimedia Appendix 2. International Classification of Diseases, Tenth Revision, Clinical Modification (ICD-10-CM) diagnosis codes indicating potential fall occurrences and comorbidities.

| **Variable** | **ICD-10-CM Codes** |
| --- | --- |
| Unintentional falls | V00.11-V00.89 W00-W15,  W16.022, W16.032,  W16.112, W16.122, W16.132,  W16.212, W16.222,  W16.312, W16.322, W16.332,  W16.42,  W16.512, W16.522, W16.532,  W16.612, W16.622, W16.712, W116.722  W16.812, W16.822, W16.832,  W16.92,  W17, W19 W18.1, W18.2, W18.3 |
| Injury to body part | S90-99 |
| Prosthetic joint | M97 |
| Complications of trauma | T79 |
| Cognition problems (e.g., dementia) | G30.0 G30.1 G30.8 G30.9 F03.90 R41.81 |
| Parkinson’s disease | G20 |
| Cardiac arrhythmia | I49.9 |
| Orthostatic hypotension | I95.1 |
| Depression | F33 F33.0  F33.1 F33.2  F33.3  F33.40  F33.41 F33.42 F33.8 F33.9 |
| Incontinence | N32.81 N39.41-39.46 N39.490 N39.491  N39.492  N39.498 |
