# Supplementary material for: Telemedicine-Based Risk Program to Prevent Falls Among Older Adults: Protocol for a Randomized Quality Improvement Trial
Source: JMIR Res Protoc. 2024 Mar 26;13:e54395. doi: 10.2196/54395 (PMC11005432; doi:10.2196/54395)
Supplement: Multimedia Appendix 3 [file resprot_v13i1e54395_app3.docx]

## Multimedia Appendix 3. Stopping Elderly Accidents, Deaths, and Injuries (STEADI) Options Trial electronic record medication flag list for medication management assessment component.

| **Variable** | **Generic Medication** |
| --- | --- |
| Tricyclic Antidepressants | Amitriptyline |
|  | Amoxapine |
|  | Clomipramine |
|  | Desipramine |
|  | Doxepin |
|  | Imipramine |
|  | Nortriptyline |
|  | Paroxetine |
|  | Protriptyline |
|  | Trimipramine |
| Antihistamines/Allergy/Cough and Cold | Azelastine |
|  | Brompheniramine |
|  | Carbinoxamine |
|  | Chlorpheniramine |
|  | Clemastine |
|  | Cyproheptadine |
|  | Dexbrompheniramine |
|  | Dexchlorpheniramine |
|  | Diphenhydramine |
|  | Hydroxyzine |
|  | Olopatadine |
|  | Promethazine |
|  | Triprolidine |
| Motion Sickness/Dizziness/Nausea | Dimenhydrinate |
|  | Meclizine |
|  | Promethazine |
|  | Scopolamine |
|  | Trimethobenzamide |
| Anticholinergics | Benztropine |
|  | Trihexyphenidyl |
| Gastrointestinal Tract Conditions | Atropine (excludes ophthalmic) |
|  | Belladonna Alkaloids |
|  | Cimetidine |
|  | Clidinium |
|  | Dicyclomine (excludes ophthalmic) |
|  | Homatropine |
|  | Hyoscyamine |
|  | Methscopolamine |
|  | Propantheline |
| Antipsychotics | Aripiprazole |
|  | Asenapine |
|  | Chlorpromazine |
|  | Clozapine |
|  | Fluphenazine |
|  | Haloperidol |
|  | Iloperidone |
|  | Lurasidone |
|  | Loxapine |
|  | Olanzapine |
|  | Paliperidone |
|  | Perphenazine |
|  | Pimozide |
|  | Prochlorperazine |
|  | Quetiapine |
|  | Risperidone |
|  | Thioridazine |
|  | Thiothixene |
|  | Trifluoperazine |
|  | Ziprasidone |
| Skeletal Muscle Relaxants | Carisoprodol |
|  | Cyclobenzaprine |
|  | Orphenadrine |
|  | Tizanidine |
| Benzodiazepines | Alprazolam |
|  | Chlorazepate |
|  | Chlordiazepoxide |
|  | Clobazam |
|  | Clonazepam |
|  | Diazepam |
|  | Estazolam |
|  | Flurazepam |
|  | Lorazepam |
|  | Midazolam |
|  | Oxazepam |
|  | Quazepam |
|  | Temazepam |
|  | Triazolam |
| Z-drugs | Eszopiclone |
|  | Zaleplon |
|  | Zolpidem |
| Opioids | Buprenorphine |
|  | Butorphanol |
|  | Codeine |
|  | Fentanyl |
|  | Hydrocodone |
|  | Hydrocodone and acetaminophen |
|  | Hydrocodone and ibuprofen |
|  | Hydromorphone |
|  | Levorphanal |
|  | Meperidine |
|  | Methadone |
|  | Morphine |
|  | Oxycodone |
|  | Oxycodone and acetaminophen |
|  | Oxycodone and ibuprofen |
|  | Oxymorphone |
|  | Tramadol |

**Notes:** This list is adapted from the 2019 update to the American Geriatric Society Beers criteria. [18]

**Abbreviations:** STEADI: Stopping Elderly Accidents, Deaths, and Injurie
