# Supplementary material for: Telemedicine-Based Risk Program to Prevent Falls Among Older Adults: Protocol for a Randomized Quality Improvement Trial
Source: JMIR Res Protoc. 2024 Mar 26;13:e54395. doi: 10.2196/54395 (PMC11005432; doi:10.2196/54395)
Supplement: Multimedia Appendix 4 [file resprot_v13i1e54395_app4.docx]

## Multimedia Appendix 4. Stopping Elderly Accidents, Deaths, and Injuries (STEADI) Options Trial educational materials distributed and reviewed to patients during assessment.

| Materials | Source | Notes on Use |
| --- | --- | --- |
| What You Can Do to Prevent Falls | [STEADI webpage](https://www.cdc.gov/steadi/pdf/check_for_safety_brochure-a.pdf):  <https://www.cdc.gov/steadi> | Provided to all patients at risk for falls in the STEADI arm. Based on patients’ screening and assessment results, the RN will discuss strategies for reducing patients’ risk of falling and review this handout with patients. |
| STEADI Footwear Handout | A CDC compendium of effective fall interventions: what works for community-dwelling older adults. 4^th^ edition:  <https://stacks.cdc.gov/view/cdc/124200> | RN reviews with patient as part of feet/footwear assessment. |
| Tai Chi for Arthritis Info Sheet | Handout created by Emory covering Atlanta organizations and companies offering online Tai Chi programs for arthritis | RN distributes to patients who pass all three Gait and Balance Tests. |
| Epley Maneuver Handout | <https://www.hopkinsmedicine.org/health/treatment-tests-and-therapies/home-epley-maneuver> | RN distributes to patients reporting dizziness. |
| Postural Hypotension – What it is & How to Manage it | <https://www.cdc.gov/steadi/pdf/STEADI-Brochure-Postural-Hypotension-508.pdf> | RN distributes to all patients with postural hypotension. |
| Check for Safety – A Home Fall Prevention Checklist for Older Adults | <https://www.cdc.gov/steadi/pdf/check_for_safety_brochure-a.pdf> | For all patients undergoing the STEADI assessment, the RN will use and provide the *Check for Safety, a Home Fall Prevention Checklist* to help the patient determine home modification that may be made to reduce their risk of falling. |
| Medication management/de-prescribing resources | Canadian Medication Appropriateness and Deprescribing Network brochures  [Sleeping pills and anti-anxiety medication brochure](https://static1.squarespace.com/static/5836f01fe6f2e1fa62c11f08/t/639c7e4223f1c933bd56dcba/1671200340392/Sleeping%2Bpills_anti%2Banxiety%2Bmeds_Sedative%2Bhypnotics.pdf)  And [Opioids for chronic non-cancer pain brochure](https://static1.squarespace.com/static/5836f01fe6f2e1fa62c11f08/t/639c7de096a05f7d725310d9/1671200228188/Opioids_Narcotics%2Bfor%2Bchronic%2Bpain.pdf) | RN distributes to patients based on their need for medication management. |

**Abbreviations:** STEADI: Stopping Elderly Accidents, Deaths, and Injuries; RN: clinical research nurse.
