# Supplementary material for: Telemedicine-Based Risk Program to Prevent Falls Among Older Adults: Protocol for a Randomized Quality Improvement Trial
Source: JMIR Res Protoc. 2024 Mar 26;13:e54395. doi: 10.2196/54395 (PMC11005432; doi:10.2196/54395)
Supplement: Multimedia Appendix 5 [file resprot_v13i1e54395_app5.docx]

## Multimedia Appendix 5. Collaboration between clinical research nurse (RN) and provider in the Stopping Elderly Accidents, Deaths, and Injuries (STEADI) Options Trial, Emory Health Services, 2020-2021.

**Abbreviations:** STEADI, Stopping Elderly Accidents, Deaths, and Injuries. EHR: Electronic health records.
