# Supplementary material for: Telemedicine-Based Risk Program to Prevent Falls Among Older Adults: Protocol for a Randomized Quality Improvement Trial
Source: JMIR Res Protoc. 2024 Mar 26;13:e54395. doi: 10.2196/54395 (PMC11005432; doi:10.2196/54395)
Supplement: Multimedia Appendix 6 [file resprot_v13i1e54395_app6.docx]

## Multimedia Appendix 6. Stopping Elderly Accidents, Deaths, and Injuries (STEADI) Options Trial evaluation design, Emory Health Services, 2020-2021.

| **Type of Measure** | **Objective** | **Indicator** |
| --- | --- | --- |
| Process  measures | Was STEADI implemented as intended? | •         Screening and enrollment indicators |
|  | Sources: | •         Assessments by video, phone, and no-shows |
|  | •         Redcap data | •         Assessment occurrence by component |
|  | •         Qualitative interviews | •         Risks identified by component |
|  |  | •         Recommendations sent to provider |
|  |  | •         Qualitative data on intervention staff and provider attitudes and beliefs about STEADI implementation |
| Short-term outcomes | Did STEADI result in anticipated interim actions that could reduce fall risk among STEADI assigned patients? | - Service utilization of assessment indicated evidence-based fall prevention strategies |
|  | Sources: | - Prescriptions for drugs that increase fall risk |
|  | - EHR information of services used within health system |  |
|  | - Survey self-reported measures of evidence-based fall prevention strategies used outside the health system. |  |
| Long-term outcomes | Did STEADI intervention reduce falls among patients assigned to the STEADI arm compared to the SOC arm? | - Occurrence of any medically treated, self-reported, and all falls over a 1-year time horizon |
|  | Sources: | - Number of medically treated, self-reported, and all falls over a 1-year time horizon |
|  | - EHR information |  |
|  | - Survey self-reported falls |  |
| Cost-effectiveness | What were the costs and what was the cost-effectiveness of this implementation? | - All-cause allowable charges within the health system and estimated costs of health services outside the health system |
|  | Sources: | - Cost and cost-effectiveness of implementation |
|  | - Time diaries by implementation staff |  |

**Abbreviations:** STEADI, Stopping Elderly Accidents, Deaths, and Injuries. SOC Standard of Care, EHR Electronic Health Records.
